# Supplementary material for: Are carbon emissions trading and green financial instruments synergistic? -Comprehensive quantitative research based on content analysis
Source: PLoS One. 2024 Mar 7;19(3):e0298601. doi: 10.1371/journal.pone.0298601 (PMC10919601; doi:10.1371/journal.pone.0298601)
Supplement: S1 Data — (ZIP) [file pone.0298601.s001.zip › Data/地级市Z_F工作报告.zip!/╣π╬≈/║╪╓▌╩╨/└┤╫╘╣1⁄2╓┌║┼í╛┬φ┐╦╩2╛▌═°í┐_2007.docx]

各位代表：

　　现在，我代表市人民政府向大会报告工作，请予审议，并请市政协各位委员和其他列席会议的同志提出意见。

一、2006年工作回顾

　　2006年是我市各项工作取得突出成绩的一年。在市委的正确领导下，在市人大及其常委会和市政协的监督、支持下，市人民政府和全市各族人民一道，全面落实科学发展观，开拓创新，扎实工作，完成了各项预期目标任务，保持了全市经济社会持续快速健康发展的良好势头，实现了“十一五”的良好开局。初步统计，全市生产总值实现190亿元, 增长13.9％，其中，一、二、三产业分别增长6.2 %、20.6%和11.7%；财政收入13.56亿元，增长20.8％; 全社会固定资产投资完成106亿元，增长27.8%。

　　───工业经济保持快速增长，质量和效益显著提高。工业园区建设取得显著成效，旺高、信都、西湾3个工业区新引进企业14家，总投资额5.1亿元；旺高工业区实现工业增加值1.03亿元，增长9.8倍。工业技改顺利推进，40项重点技改项目全部开工，完成投资10.56亿元，增长108.2%。工业产品质量、效益不断提高，已有8个工业主导产品被评为广西名牌产品。工业结构进一步优化，电子、有色金属开采加工、林产、建材、电力、制药、稀土、农副产品加工等产业初具规模，桂东电子中高压铝箔二期工程、大锰银鹤无汞碱性电池生产线、嘉禾木业中纤板等项目快速推进；吉光电子铝电解电容器项目启动建设；灵峰药业与美国东方生物技术有限公司成功重组；林浆纸一体化、珊瑚钨矿复采等项目前期工作加快推进。全市工业增加值完成73.6亿元，增长22.3%。工业增加值占生产总值的比重达38.7%，对经济增长的贡献率达58.5%。

　　───城市建设步伐加快，城市综合竞争力大幅提升。完成太白湖公园及周边地区控制性详规和城市设计以及市职教中心、嘉禾木业板厂等16个重大项目的详细规划。大力实施总投资达46亿元的25个城市基础设施建设项目，完成投资6亿多元，建设路、贺州大道等城区道路改造项目建成投入使用，汇豪国际城、远东国际城、香港商贸城等房地产项目加快建设，管道燃气、垃圾处理厂、市汽车客运中心和一批专业市场建设进展顺利，人民防空工程全面启动，城市功能不断完善。实施“城乡清洁工程”、开展“争当文明市民”活动深入推进，城市长效管理机制逐步完善，“五乱”现象整治成效显著，市容市貌明显改观。县城、重点镇建设也取得新成绩。

　　───新农村建设扎实推进，农业和农村经济稳步增长。优势农产品标准化、规模化和基地化建设深入推进，全市建成45万亩无公害标准化生产示范基地和189个标准化养殖示范场、9个生态养殖小区，速生丰产林面积达到64.8万亩。品牌农业快速发展，八步区被国家林业局命名为“中国李子之乡”，“富江牌”脐橙被农业部评定为2006年度中国名牌农产品，另有7个农产品被评为广西名牌产品或广西优质农产品。农业企业化取得新突破，新增2家自治区级重点农业龙头企业，新增规模以上农业企业35家，新增农民专业合作协会8个。县域经济蓬勃发展。新增劳务输出3.71万人，劳务经济收入达36.15亿元，增长15%。农村基础设施不断完善，实施8个通乡油路项目共202公里，完成88处冬修水利工程建设，新建11处农村饮水解困工程。30个新农村建设试点村的工作顺利推进。全市农业总产值实现89.8亿元，增长6.2%。

　　───商贸旅游市场繁荣活跃，第三产业快速发展。旅游产业加快发展，姑婆山、紫云旅游景区分别评定为国家4A级、3A级景区，黄姚古镇被评为“中国最值得外国人去的50个地方”金奖景区和中国最具有旅游价值古城镇。旅游宣传促销力度加大，客源市场进一步拓展。全年接待国内外游客309.5万人次，增长12.4%；旅游总收入15.7亿元，增长21%。“万村千乡”市场工程初见成效，新建、改建标准“农家店”150个。现代服务业快速发展，城乡市场繁荣兴旺。全社会消费品零售总额39.5亿元，增长14.5%。房地产业完成交易额3.78亿元，增长18.7%。金融业稳步发展，金融秩序良好。非公有制经济发展取得新成绩。

　　──固定资产投资快速增长，重点项目建设成效显著。全社会固定资产投资额首次突破百亿元大关，保持了投资对经济增长的强劲拉动。百项工程大会战和交通基础设施建设大会战取得新成效，全年新开工投资规模50万元以上项目772个，完成投资37亿元；续建项目479个，完成投资42亿元；竣工项目517个，完成投资24亿元；桂梧高速公路平乐至钟山段、323线莲塘至鹰扬关二级路建成通车，同古至马江段完成工程量的53%；洛湛铁路贺州段完成总投资额的71%；贺州港信都作业区等一批设施项目开工建设，源头至灵峰二级路水泥路面改造工程正在实施；广贺高速公路贺州段控制性工程动工建设。永贺高速公路、贺州火电厂和广州经贺州至贵阳快速铁路项目的前期工作正在加紧推进。

　　──改革开放不断深入，发展活力进一步增强。行政管理体制改革继续深化，事业单位机构和人事制度改革试点工作稳步推进。粮食企业及其流通体制改革基本完成。兽医管理体制改革、供销社系统企业改革顺利推进。财政改革取得成效，市本级财政国库集中支付系统启动运行，政府采购进一步规范。顺利完成9户国有企业产权制度改革。积极参与泛珠三角、泛北部湾和中国—东盟“一轴两翼”多区域合作，组织参加东盟各国投资合作洽谈会、第三届中国—东盟博览会等重大招商引资活动，派出文化代表团赴泰国、柬埔寨和台湾演出联谊、洽谈项目。与广东佛山、浙江衢州、湖南永州及柳州铁路局交流合作进一步加强。先后派出5批招商小分队奔赴广东、福建、浙江等地开展驻点招商和专题招商，成功举办市第三届农产品交易会、首届房展会以及赴广东开展电子、家具、稀土三大产业招商推介活动。全年共新引进内资项目665个，合同金额147.78亿元，利用外来资金到位70.84亿元，增长23.23%；引进外资项目15个，实际利用外资1304.4万美元，增长107.1%。对外贸易略有增长，外贸出口总额达9310.9万美元。

　　──各项社会事业全面发展，人民生活明显改善。科技投入力度不断加大，对经济的支撑引领作用进一步发挥。农村义务教育得到加强，“两基”成果进一步巩固提高；高等教育和职业技术教育加快发展，贺州学院申办成功，招录了第一批本科生入校学习；市职业教育中心建设进展顺利。自治区级先进文化市创建活动扎实推进，舞台艺术创作取得新成绩。新闻出版事业获得新发展。广播电视村村通建设成果得到巩固拓展，市、县城区有线电视数字化整体转换工作顺利完成。公共卫生、基本医疗服务体系、重大疾病防控体系建设取得新进展，新型农村合作医疗试点工作得到巩固提高。市体育中心场馆建设顺利推进，群众性体育蓬勃开展，竞技体育取得新成绩。建立完善农村计划生育利益导向机制工作获新突破，获得广西唯一的“全国对农村计划生育家庭实施奖励扶助政策先进市”称号，人口自然增长率控制在8‰以内。全市城镇居民人均可支配收入8619元，增长14.7%；农民人均纯收入2682元，增长14.1%。

　　──精神文明建设卓有成效，社会保障体系逐步完善。“桂东文明绿洲”创建活动广泛开展，未成年人思想道德建设得到加强，湘粤桂边界三市区域性精神文明共建活动扎实推进。全国双拥模范城创建活动深入开展。“五五”普法全面启动。六项“民心工程”建设成效显著，五保村建设和民政管理机制进一步完善；整村推进贫困村扶贫开发取得实效，解决4007人贫困人口的温饱问题，低收入贫困人口脱贫8052人；完成残疾人危房改造1562户；就业、再就业和社会保障工作不断加强，制定出台了农村居民最低生活保障、廉租房、经济适用房等惠民利民政策，各项社会保险参保人数均超额完成年度任务，新增城镇就业12169人，下岗失业人员再就业4118人，城镇登记失业率为3.49%。“平安贺州”创建活动有效开展，食品和药品监管、公共安全和安全生产监管进一步加强，治安和刑事案件发案率分别下降45.2%和1.9%，各类安全事故起数、死亡人数、受伤人数和直接经济损失分别下降31.3%、26.9%、25.9%和19.1%。国防动员、民兵预备役等工作继续加强，妇女儿童、老龄、残疾人事业以及人事、机构编制、工商管理、技术监督、出入境检验检疫、审计、统计、物价、民政、民族、宗教、外事、侨务、对台、防震减灾、气象、修志、档案、工会、住房公积金等工作取得新进步。

　　──政府自身建设有效推进，依法行政水平得到提高。顺利完成了市、县(区)、乡（镇）三级政府换届工作。进一步完善政府决策机制，畅通市长热线、市长电子信箱等渠道，对与群众利益密切相关的事项实行公示、听证等制度，广泛听取意见。大力推进行政审批制度改革，加快市政务中心提速工程建设，清理取消30个单位依据不足、时效过期的审批事项168项。政务公开有效推进，电子政务建设有新进展。严格行政执法责任制，深入开展机关效能建设，机关办事效率和服务水平不断提高。认真落实廉政建设责任制，深入开展治理商业贿赂工作，查处了一批违纪违法案件。

各位代表，过去的一年，我们把做好当前和谋划长远有机结合起来，创新发展思路，完善工作措施，有效推进了各项工作。有几条成功的经验值得总结和发扬：一是坚持与时俱进，科学发展。把上级精神和贺州实际结合起来，千方百计加快发展，更加注重经济结构的优化调整和经济社会的协调发展，走有贺州特色的发展路子，提高经济增长和社会发展的质量和效益。二是坚持重点突破，带动全局。突出抓好基础设施建设、工业发展、城镇建设、项目实施和招商引资、为民办实事等重点，加快发展园区经济、通道经济、特色经济和循环经济，实现了新突破，推动了工作的全面开展。三是坚持深化改革，扩大开放。以改革增活力，以开放促开发，推进经济主体向多元市场主体转变，产业发展向资源原材料深加工、产业聚集转变，发展模式向集约型、科技型转变，投资方式向大范围招商引资转变，政府职能向服务型转变。四是坚持以人为本，执政为民。始终把解决人民群众最关心、最直接、最现实的利益问题作为工作的出发点和落脚点，更加关注民生问题，深入实施和拓展六项“民心工程”，让人民群众最大限度地共享改革发展成果。五是坚持维护稳定，和谐发展。切实把科学发展与和谐发展结合起来，加强民主法制建设，及时协调各种利益关系，有效化解社会矛盾，在社会稳定中推进改革与发展。

　　各位代表，过去的一年，我市经济和社会发展取得了显著成绩。这是自治区党委、自治区人民政府亲切关怀，市委坚强领导的结果，是全市各级人大和人民政协监督支持的结果，是全市各级干部和各族人民克服困难、真抓实干、开拓进取的结果。我代表市人民政府，向全市各条战线上辛勤劳动、做出贡献的广大劳动者和建设者，向给予我们监督支持的各级人大代表、政协委员，向各民主党派、工商联、无党派人士、各人民团体，向驻贺解放军和武警官兵，向所有关心支持贺州建设的社会各界人士和国内外朋友表示崇高的敬意和衷心的感谢！

　　在肯定成绩的同时，我们也必须清醒地看到发展中面临的困难和问题，主要是：经济总量小，产业结构不够合理，财力仍然薄弱；农业基础不够牢固，农民增收渠道不多，增收难度大；强优企业不多，重大产业项目少；部分项目建设受征地拆迁影响，进展不够快；固定资产投资增幅在前几年高速增长的基础上出现较大幅度的回落；进出口增长较慢；机关工作作风和效能建设有待加强；就业和社会保障、扶贫开发、公共安全等方面还需要做大量工作；等等。这些困难和问题，我们要在今后的工作中采取措施加以解决。

二、2007年主要目标和工作要求

　　今年是建设富裕和谐新贺州的关键之年。政府工作的总体要求是：以邓小平理论和“三个代表”重要思想为指导，认真贯彻自治区第九次党代会、市第二次党代会精神，全面落实科学发展观，按照“五业并举，工业优先，夯实基础，做大总量”的要求，突出发展园区经济、通道经济、特色经济、循环经济，加快推进工业化、城镇化、农业企业化，巩固提高商贸旅游业，着力统筹城乡发展，着力调整经济结构和转变增长方式，着力加强资源节约和环境保护，着力推进改革开放和全民创业，着力促进社会发展和改善民生，努力实现经济又好又快发展，推动富裕和谐新贺州建设，以优异成绩迎接党的十七大胜利召开。

　　建议今年全市经济社会发展的主要预期目标为：全市生产总值增长12%以上；单位生产总值能耗降低2.1%，主要污染物排放总量减少3.2%；财政收入增长15%；全社会固定资产投资增长20%；全社会消费品零售总额增长12%；外贸出口总额增长12%；城镇居民可支配收入增长10%；农民人均纯收入增长11.5%；城镇新增就业人数增长8%。

　　今年经济社会发展主要目标，是在综合考虑各种因素的基础上，本着积极稳妥的原则提出的。节能、减排等指标是约束性的，生产总值增长速度等其他指标是预期性、指导性的。作为后发展地区，我市与全国、全区发展差距还比较大，必须在优化结构、提高效益、降低消耗的基础上，进一步提高经济增长速度，能快就不要慢，努力赶上全国全区的发展步伐。

　　为全面实现今年的经济社会发展各项目标，必须把握好以下五个方面：一是把加快发展放在第一位，把科学发展观作为长期坚持的重要指导思想，促进速度、质量、效益相协调，投资、消费、出口相协调，人口、资源、环境相协调，真正做到又好又快发展。二是把推进结构调整作为提高经济整体效益的关键，把工业化、城镇化作为加快发展的主导方向，把加大投资和招商引资力度作为增强发展后劲的根本保障，积极促进三次产业、城市与农村发展、投资与消费、经济发展与环境保护良性互动。三是遵循经济发展规律和市场法则，积极探索和研究从根本上解决问题的办法，进一步消除影响经济社会发展的制约因素，努力创造良好发展环境，增创发展新优势。四是把改善民生作为第一责任，认真解决好人民群众最关心、最直接、最现实的利益问题，保持社会和谐稳定。五是转变政府职能，改进机关作风，弘扬艰苦奋斗、干事创业、真抓实干的精神，逐项工作、逐件事情抓好落实，不断实现工作的新突破，开创发展的新局面。

三、努力实现又好又快发展

　　始终坚持把加快发展作为富民强市的第一要务，统筹兼顾，突出重点，下大力气抓好各项工作，在更高层次上实现又好又快发展。

（一）培育壮大工业支柱，强化工业经济的主导地位

　　突出抓好“八大产业”、“三大重点”。立足现有产业基础和资源优势，大力培育和发展优势特色产业，使之成为带动工业经济发展的支柱产业，形成产业集群。力争全市工业增加值完成90亿元，全部工业投资完成48亿元，分别增长18%和20 %。

围绕电子、有色金属开采加工、林产、建材、电力、制药、稀土、农副产品加工等八大优势产业和电子、有色金属开采加工、林产三大重点产业，落实新引进项目，加快在建项目进度，建好一批新工业项目。电子产业重点是加快吉光电子公司铝电解电容器项目建设，引进一批电子铝箔深加工企业，发展电子铝箔上下游产品；林产工业重点是推进林浆纸一体化项目，抓好嘉禾木业年产24万立方米中纤板、10万套家具项目建设，推动家具制造业发展；有色金属开采加工业重点是发展壮大平桂飞碟、金易冶炼,抓好珊瑚钨矿复采;建材工业重点是抓好英格瓷、梅林化工等重钙超细粉加工企业建设，形成粉体加工产业集群；稀土产业重点是抓好金源稀土公司年产1000吨钕铁硼永磁材料项目建设，发展稀土矿开采、高纯稀土深度分离和稀土应用深加工；电力工业要进一步整合资源，推进水电开发，完善供电网架，积极推进火电厂建设，做大电力产业；农副产品加工业要围绕马蹄、茶叶等优势产品加工，延长产业链，带动农民增收。

　　强化工业园区建设。根据我市发展条件不断改善、东部产业转移步伐加快的形势，把工业园区作为今后我市工业发展的主力军来抓好。抓紧完善旺高、信都、西湾工业区的规划和各县（区）工业集中区的规划以及八步至钟山二级公路沿线工业经济带的控制性详规。进一步理顺工业园区的管理体制，充分利用好上级的扶持政策和资金，创新完善园区的建设、运营和利益分配机制，引进有实力的投资商合作，切实加强工业园区的基础设施建设，扩大园区规模、完善设施条件，提高项目承载能力和配套服务水平，吸引更多的企业和产业向园区聚集。

　　加大工业技改力度。巩固提高现有工业水平，培育发展强优企业。重点抓好桂东纸业年产10万吨高级文化纸、灵峰药业软胶囊中成药技改扩产、西麦食品公司燕麦生产线改造、桂东水电公司中高水头水轮机组产品技改、真龙彩印等技改项目，力争全年技改投资完成9.2亿元。加强指导，努力提高工业企业的经营管理水平，增强综合实力和竞争力。大力打造贺州名牌，力争全年有2-3个工业产品获广西名牌产品称号。

　　注重民营中小企业的发展。建立中小企业服务体系，引进或成立中小企业担保公司，探索多种融资渠道、多种担保形式，努力为中小企业和民营经济提供信用担保支持，促进民营工业快速发展。

（二）以中心城市和县城建设为重点，加快推进城镇化

　　突出抓好中心城市建设。大力实施“五年创新城”城市建设方案。完善城镇规划体系，完成旧飞机场、火车站站前广场及西约街、新宁街片旧城区改造等控制性详规和城市照明、排水、排污、电力专项规划。加大城区交通、管网、绿化、供水、人防、安居等基础设施建设力度，着力推进市区“一江两岸三片区”规划建设，即推进贺江及沿江两岸和城东片区、城西片区、城南片区建设，加快建设市文化会议中心、垃圾处理厂、污水处理厂、龟石供水二期工程、管道燃气、贺州火车站、汽车客运中心、职教中心、体育中心、家居销售中心等一批市政项目，抓好经济适用房、汇豪国际城等房地产项目建设，扩大城市规模。积极发展城市公共交通，改善市民出行条件，完善城市功能，提升城市品位。

　　深入实施“城乡清洁工程”，开展“争当文明市民”活动。全面落实自治区人民政府关于实施“城乡清洁工程”的决定，坚持重点整治和日常管理相结合，重点治理“五乱”现象，抓好专项治理的巩固提高，突出治理城镇脏乱差面貌。进一步加强市区垃圾收集和集中处理设施建设，加大城市综合执法力度，建立完善市容市貌长效管理机制。进一步强化市民的城市意识，全面提升城市经营和管理水平。

　　加快县城和重点镇发展。大力抓好钟山、富川、昭平3个县城的规划和建设，完善和规范城镇户籍管理、征地拆迁、环境卫生等各项制度措施，积极鼓励和引导农民进城安居创业，加快向小城市的方向发展。注重完善县城基础设施，年内各县城启动污水处理厂和垃圾处理厂的规划建设。切实抓好25个重点镇建设，培育发展高速公路、铁路、高等级公路沿线的城镇带。

（三）进一步加强“三农”工作，扎实推进社会主义新农村建设

　　大力发展现代农业。进一步推进优势农产品的标准化生产、规模化经营和区域化布局，加快优势产业带建设，做大做强无公害蔬菜、脐橙、烟叶、马蹄、桑蚕、食用菌、茶叶、梅李、松脂林、速丰林、瘦肉型猪、优质肉牛、优质家禽、名优水产品等产业，促进资源型大工业和现代农业紧密结合。推进农业企业化经营，加大对农业龙头企业的培育和扶持力度，力争全年新增规模农业企业30家以上、农民专业合作协会5家。大力推广农业实用增产技术。认真贯彻中央购机补贴惠农政策，促进农业机械化进程。加快发展品牌农业和生态农业，全面推进无公害农产品行动计划，加强农产品质量安全体系建设，强化农产品全过程质量安全管理，提高农产品的质量和档次，创建优质农产品品牌。全年组织申报名优农产品品牌3—5个，新增农产品注册商标10个，力争富川成为中国脐橙之乡。同时，做好我市名特优产品地理标志保护申报工作。

　　加快发展县域经济。把发展县域经济作为新农村建设的着力点，统筹城乡产业布局，加快县城工业集中区的建设，引导劳动密集型、资源加工型产业向县域集聚，鼓励农产品加工业向主产地集中，加快把农产品加工业培育成为县域经济的主导产业。大力开展农村劳动力转移培训，注重培养各类农村实用人才，着力提高农民的就业和创业能力，全年培训农村劳动力11万人次。扩大劳务输出规模，增加农民工资性收入，全年新增劳务输出2.5万人。

　　全面推进新农村建设。搞好新农村建设总体规划，推进新农村建设试点工作，加快建立合理、稳定、有效的支农资金投入机制，突出抓好农村道路、水利、人饮安全工程、沼气推广以及教育、文化、体育、卫生、厕改等公共设施建设。年内修建贫困村屯道路110条312公里，建设人饮安全工程40处，修建沼气池1.7万个。加强农村生态建设，搞好村庄规划和植树绿化，治理农村人居环境，改善农村生产生活条件。

　　深化农村综合改革。认真落实各项支农惠农政策，巩固农村税费改革成果，着力推进乡镇机构、农村义务教育和县乡财政管理体制等为主要内容的农村改革试点，建立精干高效的农村行政管理体制、覆盖城乡的公共财政制度和政府保障的农村义务教育体制，促进农民减负增收和农村公益事业的发展。

（四）巩固提高商贸旅游业和服务业，推动第三产业加快发展

　　发展提升商贸物流业。进一步加大商贸物流业的规划、建设和管理力度。编制全市商贸流通业发展总体规划，建立和完善商业网点审查审批制度。抓好汽车农机销售中心、农资市场、建材市场、水果批发市场等专业市场的规划建设；在市区新建和改造4个农贸市场，改善和提升商贸物流设施水平。引进有实力的商贸企业发展新型商贸业态，扩大商贸业规模。加强商贸流通服务业管理，引导商贸企业规范经营、拓展业务、提升水平、做大做强。推进“万村千乡”市场工程和“新网工程”，大力开拓农村市场，推进超市、连锁商业网点向农村市场延伸。

　　大力发展旅游业。加大力度，完善措施，积极争创全国优秀旅游城市。加大旅游业投入，完善旅游基础设施尤其是通往景区道路的建设，突出加强姑婆山旅游区和黄姚景区的建设，进一步加强紫云景区、碧水岩圣景山庄、客家围屋、农业观光园、秀水状元村的建设与改造。进一步规范旅游市场管理，提升旅游服务档次和水平。加大旅游市场推介和促销力度，积极开拓国际、国内旅游市场，力争接待国内外游客和旅游总收入有较大幅度增长。

　　积极发展各种服务业。大力探索、运用市场规律运作好各类会展活动，努力打造农产品交易会等区内外具有影响力的会展品牌，培育发展会展业。大力支持金融保险业加快发展，力争更多的金融机构升格，与我市的发展适应配套。积极发展房地产、信息服务、中介服务等服务业，大力发展各类便民、利民的社区服务业，完善和提升服务业整体结构。

（五）着力推进项目建设，保持投资对经济增长的强劲拉动

　　加强重点领域建设。继续大力实施百项工程大会战和交通基础设施建设大会战，加快推进桂梧高速公路同古至马江段、广贺高速公路、洛湛铁路贺州段和昭平至蒙山黄村、八步经黄姚至昭平二级公路建设以及源头至灵峰二级公路二期改造。全面开工建设列入“十一五”规划的尚未开工的16条通乡油路和水泥路，加快贺州港信都作业区建设，完善交通路网结构。积极推进重大项目新开工，力争今年新上投资规模1000万元以上的项目80个以上，总投资122亿元以上，完成投资27亿元以上。加快在建项目建设步伐，抓好108个续建项目，力争完成投资58亿元以上。同时，抓好其他项目建设。确保全社会固定资产投资完成127亿元，力争突破130亿元。

　　做好项目前期工作。根据国家宏观政策走向和投资重点领域，在基础设施、特色产业、生态环境、节能降耗、社会公益等领域，规划一批重大项目，争取有更多项目列入自治区和国家规划。做好一批重大项目前期工作，重点推进和配合做好永贺高速公路贺州段、贺州火电厂、广州经贺州至贵阳快速铁路、林浆纸一体化等重大项目的前期工作。

　　着力解决项目建设中存在的具体问题。制订和完善征地拆迁工作顺利推进、群众利益得到实现和维护的机制，努力解决好征地拆迁难的问题。统筹安排好项目建设用地，优先考虑和确保市统筹推进的重大项目和其他急需项目建设用地指标。加大盘活存量土地力度，做好年度土地利用计划和土地储备工作。运用市场机制筹措资金，促进投资来源多样化。积极争取国家和自治区资金支持，稳定增加财政资金投入。大力培育新型投融资主体，引导民间投资进入基础设施和社会事业项目建设。有效开展政企、银企合作，争取银行扩大授信额度，增加贷款规模。

（六）加强区域合作，提高对外开放水平

　　积极承接东部产业转移。加强东部产业转移趋势的研究，制定和完善鼓励措施，落实好国家、自治区和我市各项鼓励产业发展的优惠政策，降低投资门槛和成本，通过参与区域合作、搭建合作平台等多种方式，促进东部产业加快向我市转移，把我市打造成为承接东部转移产业的重要基地。

　　扩大区域合作。认真研究区域合作的形势，找准切入点和活动载体，采取有效措施进一步加强与珠三角核心区、泛北部湾、长三角、大西南和东盟国家合作的区域经济合作，提升区域合作的规模和质量。抓紧落实与广东佛山、浙江衢州、湖南永州及柳州铁路局的经贸合作事项，认真履行各项合作协议，推动重点合作领域项目抓出实效。

　　强化招商引资工作。按照“融入粤港澳、拓展闽苏浙、挺进东南亚”的思路，创新招商引资方式，完善招商引资机制，优化招商引资环境，明确招商引资重点，落实招商引资责任制，参与和办好各项重大招商引资活动，力争签订一批新的国际国内合作项目。高度重视和抓好利用外资和“百企入桂”工作，有针对性地做好有影响的优势企业的招商引资工作。完善部门分工协作机制和跟踪服务机制，建立健全招商引资定期通报工作制度，促进签约项目落实，提高合同履约率、项目开工率和资金到位率。

　　努力扩大外贸出口。进一步优化出口产品结构，提高出口产品的质量和效益，扩大锡和锡制品、金属硅、电子、生物制品、玩具、农产品、陶瓷、大理石等优势产品出口的比重，增强出口创汇能力和市场竞争能力。

（七）加快发展民营经济，努力增强经济发展活力

　　加快发展民营经济。优化政务环境，落实和完善加快民营经济发展的相关政策措施，贯彻平等准入、公平待遇原则，积极发展内外资企业，依法保护个体民营企业的合法权益，营造公平竞争的市场环境和政策环境。建立完善民营企业信息交流平台。努力解决民营企业实际市场准入门槛高、融资难等问题，支持和帮助有条件的民营企业扩大规模、提升层次，引导民营经济理性投资、健康发展。大力宣传民营经济的先进典型和经验，大张旗鼓地表彰先进民营企业家，为民营经济发展营造良好的舆论氛围和社会环境。

　　大力推进全民创业。研究并建立完善创业支持和服务体系，积极鼓励各行业放开创业，鼓励外出务工农民回乡创业。加大各类创业人才培育力度，进一步激活各类创业主体。加强自主创业宣传力度，在全社会弘扬创业精神和创业文化，激发创业热情，发挥创业潜能，营造良好的创业舆论环境。

（八）加强资源节约和环境保护，加快建设资源节约型、环境友好型社会

　　大力加强环境保护。实施污染物排放总量控制和排污许可证制度，强化对污染源管理和监控，严格控制污染物排放总量，禁止超标和无证排污。加强水源地保护和重点流域环境污染综合整治，提高水污染和大气污染防治能力。严禁在城区和郊区采石开矿，保护自然生态环境。

　　突出抓好重点领域的节能减排。大力推广节能环保技术，淘汰高能耗、高污染、低效益的工艺、技术、设备和产品，利用先进技术改造高耗能、高污染企业。大力发展循环经济，推行清洁生产，推进节能型产业发展，从严控制新开工高耗能项目。着力抓好工业节能，强化建筑节能、交通节能和商用、民用节能。鼓励居民和农民积极利用太阳能。加快普及农村户用沼气，抓好农村新能源建设。

严格执行节能减排目标责任制。健全节能环保工作实绩评价考核机制和问责制，将节能减排指标纳入经济社会发展综合评价和年度考核体系，根据分解目标和考核指标，对节能减排目标完成情况实行逐级考核，努力实现节能降耗和污染物减排的约束性目标。

　　节约集约用地。全面落实中央和自治区关于严格土地管理、加强土地调控的各项措施，实行严格的土地管理制度。认真执行土地利用总体规划和年度计划，统筹安排好建设用地。抓紧完善和严格执行节约和集约用地标准，提高土地利用效率和集约化程度。认真执行工业用地出让最低标准，落实建设用地税费政策，规范土地出让收支管理。严格土地管理责任制，加强耕地特别是基本农田保护，严肃查处各类土地违法违规行为。

（九）狠抓增收节支，确保财政收入稳定增长

　　大力培植财源。充分利用财政税收杠杆，积极培植、扶持税收主体和骨干税源, 把开源的重点放在工业和项目投资上，把挖潜的重点放在第三产业上，支持一批重点财源项目的建设和发展，培植利税大户。搞好国有资产经营，盘活闲置资产，做好经营城市和以地生财文章，发展创税农业，构建多元化的财源体系。依法强化税收征管，完善征管手段和控管体系，严厉打击偷、逃、骗税行为，切实做到应收尽收。改革非税收入管理制度，拓宽非税收入管理范围，严格收支两条线管理。

　　优化支出结构。财政支出更加注重社会和谐，更加关注民生民本，努力扩大公共财政对农村的覆盖面。依法优先保证各项法定支出的需要，保证工资的正常发放和机关的正常运转需要，保证各项政策性和重点支出的需要。严格控制一般性支出，压缩一切非必要的和不合理的开支。继续集中财力保重点、办大事，集中使用教育、卫生、文化、体育、科技和科普等公共基础设施的财政资金，形成资金合力，提高财政支出效益。推进国库集中收付制度改革、县乡财政管理体制改革、行政事业单位国有资产管理改革，完善政府采购制度，加快部门预算改革步伐，强化预算约束。建立健全政府债务管理制度，加强政府债务管理，防范政府债务风险。

　　加强金融工作。加强与金融部门的沟通协调，积极向银行推介优势产业、重点项目和强优企业，鼓励各家银行扩大贷款规模。继续推进农村信用社改革。积极向上级争取金融机构升格，提高金融服务水平，发挥金融对地方经济发展的促进作用。

四、大力推进和谐贺州建设

　　把构建社会主义和谐社会摆在更加重要的位置，着力促进社会发展，着力解决民生问题，着力维护社会稳定，不断促进社会和谐。

（一）大力发展各项社会事业

　　加快科技创新步伐。深入实施第三轮科技创新计划，做好科普工作，加强县乡科技服务。加快建立和完善以企业为主体、市场为导向、产品创新为核心、产学研相结合的技术创新体系。整合科技资源，组织技术攻关，在特色优势产业和领域形成一批拥有自主知识产权的核心技术和产品。

　　优先发展教育。全面落实农村义务教育经费保障机制和校舍维修改造长效机制，巩固提高“两基”成果，确保通过国家验收。加强薄弱学校建设，促进教育均衡发展。实施中小学校现代远程教育项目，推进教育信息化、现代化建设。加快普通高中资源建设，扩大办学规模，提高教学质量。大力发展职业教育，加快市、县（区）职教中心建设。推进贺州学院建设,加快高等教育发展。

加强人才队伍建设。加强党政人才、企业经营管理人才、专业技术人才队伍建设，加大人才资源开发力度。大力培养专业化、有技能的产业工人和农村实用人才。突出抓好人才小高地和高技能人才队伍建设，深入实施“两广人才互动”工程。推进贺州人才市场建设，规范人才市场管理，努力营造人才辈出、人尽其才的社会氛围。

　　加快发展卫生事业。加快建设覆盖城乡居民的基本卫生保健制度，推进三县新型农村合作医疗试点工作，争取明年全市实现新型农村合作医疗，积极推进农村贫困家庭医疗救助，着力缓解农民看病贵、看病难问题。理顺职业卫生监管体制。加强公共卫生体系建设，提高重大疾病预防控制能力和医疗救治水平。加强食品、药品安全监管，确保食品药品安全。全面改善卫生基础设施条件，加快市人民医院综合楼、桂东人民医院综合楼建设步伐。

　　加强人口和计划生育工作。坚持依法行政、思想政治教育与利益导向相结合，千方百计稳定低生育水平，提高出生人口素质。综合治理出生人口性别比偏高问题。不断完善流动人口管理服务体系。逐步构建养老服务体系。从财政、基础设施、人力、科技等方面加大投入，促进人口和计划生育事业持续健康发展。

　　积极发展文化等各项事业。坚持社会主义核心价值体系，大力发展文化事业。在文学艺术、新闻出版等领域生产更多体现民族特色、反映时代精神的文化精品。加快发展公益性文化事业，建设一批公共文化设施。继续创建自治区级先进文化市，做好昭平县创建全国文化先进县的指导和协调工作。加强广播电视基础设施建设。进一步开展“扫黄打非”工作，维护知识产权。大力发展体育事业，办好市第二届体育运动会。关心和支持老龄、妇女、儿童、残疾人等事业。继续做好人事、机构编制、工商管理、技术监督、审计、民族、宗教、外事、侨务、对台、统计、民政、物价、气象、档案、修志、工会、住房公积金等工作。

（二）着力解决涉及群众切身利益的问题

　　深入实施“民心工程”。认真落实自治区人民政府提出今年为民办的十件实事，进一步丰富我市六项“民心工程”内涵，提升水平。巩固提高“五保村”建设水平，进一步改善五保老人生活条件。扎实抓好扶贫助残“一揽子”工程、广播电视“村村通”、计划生育奖励扶助、城市“零就业”家庭、特困复员退伍军人帮扶、城市低收入贫困居民住房改造、农村饮水安全、贫困大学生资助等工作，落实农村特困群众最低生活保障、城镇廉租房制度和对农村义务教育阶段贫困寄宿生生活补助的政策，多为群众办实事，着力解决事关群众切身利益的突出问题。

　　千方百计扩大就业。进一步完善劳动者自主择业、市场调节就业、政府促进就业的机制，积极开发新兴服务业和公益性就业岗位。统筹做好城镇新增劳动力就业、下岗失业人员再就业和农村富余劳动力转移就业，加强大中专毕业生、退伍军人、失地农民的就业指导和服务，优先解决“4050”人员和城镇“零就业家庭”的就业问题。全年新增城镇就业1万人，年末城镇登记失业率控制在4%以内。

抓好扶贫开发工作。全面启动第二批40个贫困村的整村推进扶贫开发，着力抓好基础设施建设、产业开发、改善生产生活条件和扶贫培训，力争农村未解决温饱贫困人口减少8500 人，低收入贫困人口减少1万人。切实抓好库区移民搬迁安置，认真落实大中型水库移民后期扶持政策。

　　加快社会保障体系建设。继续巩固基本养老金按时足额发放成果，进一步扩大基本养老、失业、医疗、工伤、生育等各类社会保险覆盖面。健全完善城市最低生活保障制度。完善基本养老保险基金市级统筹制度，加强社会保险基金征缴和监管，增强保障能力。健全社会保险、社会救助、社会福利和慈善事业相衔接的社会保障体系。加强防灾减灾救灾工作。

　　努力争创全国双拥模范城。加强国防动员、民兵预备役建设和人防工作。全力做好全国双拥模范城检查验收迎检工作，争创全国双拥模范城。

（三）加强民主法制建设和维护社会稳定

　　加强精神文明建设和民主法制建设。坚持以“八荣八耻”为主要内容的荣辱观规范行为、引领社会风尚，高度重视未成年人思想道德建设和大学生的思想政治教育。以“桂东文明绿洲”创建活动为载体，广泛开展文明城市、文明村镇、文明社区和文明行业创建活动。进一步巩固和扩大基层民主，大力推进政务公开、村务公开、厂务公开，完善村民自治、社区民主管理和企业民主管理。全面推进依法治市，深入开展“五五”普法教育，强化执法监督，维护司法公正。自觉接受人大及其常委会的依法监督，认真执行人大及其常委会的各项决定和决议。加强与人民政协的协商，接受人民政协的民主监督。认真办理好人大代表议案、建议和政协委员提案。广泛听取各民主党派、工商联和无党派人士的意见，发挥工会、共青团、妇联等人民团体的桥梁纽带作用。

　　强化安全生产工作。强化企业安全生产主体责任，健全安全生产监督管理体制，严格执行重大安全生产事故责任追究。继续开展安全生产专项整治，着力解决烟花爆竹、危险化学品、道路交通等事故多发领域和行业的问题，预防和遏制重特大事故发生。

　　维护社会稳定。加强社会治安综合治理，健全社会治安防控体系，坚持打、防、控、管相结合，依法严厉打击各种犯罪活动，切实保障人民群众生命财产安全。推进“平安贺州”建设，深入开展“三无村屯（社区）”创建活动，促进社会安定和谐。进一步做好矛盾纠纷尤其是土地、水利和山林“三大纠纷”的调处工作，妥善处理群体性事件。加强基层公安派出所、司法所、人民法庭建设。

五、着力建设人民满意的政府

　　按照“为民、务实、清廉”的要求，加强政府自身建设，着力建设法制政府、服务政府、责任政府和效能政府，更好地为人民服务。

（一）以建设对人民高度负责、人民满意政府为目标，不断转变政府职能。推进行政管理创新，强化政府的经济调节、市场监管、社会管理和公共服务职能，在推进经济又好又快发展的同时，真正把工作的着力点放在开展平安建设、解决民生问题、发展社会事业、打造宜居环境等关系人民群众切身利益的民心工程上来，多做打基础的事，多做起长远作用的事，推进富裕和谐新贺州的建设。

（二）以提高政府执行力和公信力为重点，积极推进依法行政。认真贯彻《全面推进依法行政实施纲要》，健全科学民主决策机制，进一步完善重大问题集体决策、专家咨询、社会公示和听证制度，完善政务公开和信息发布制度，提高决策透明度和公众参与度。继续完善行政复议制度，建立权责统一的行政执法责任制，做到有权必有责，用权受监督，侵权要赔偿，违法要追究，切实克服交叉执法、多头执法、执法不公问题，提高政府的执行力和公信力。

（三）以深入实施“城乡清洁工程”为载体，切实转变机关作风，切实提高行政效能。结合实施“城乡清洁工程”，扎实开展以干部作风整顿为重点的“机关效能建设年”活动，切实加强以行政效能建设为核心的机关行政效能、政府执行力和政务环境建设，着力抓好长效机制建设，严格实行首问责任制、全面推行限时办结制、问责制，积极推进政府职能转变，努力创造良好的发展软环境。积极推进电子政务建设，深化行政审批制度改革，强化行政审批统一监管，切实提高办事效率和服务质量。大力精简会议和文件，简化公务接待，确保各级政府公务员集中精力到一线抓落实。

（四）以公务员队伍建设为保障，进一步强化从严治政。落实党风廉政建设责任制，进一步完善惩治、预防腐败体系和治理商业贿赂的长效机制，加强对权力的制约和监督，进一步规范建设工程招标投标、经营性土地出让、政府采购、产权交易等领域的制度建设，严惩为政不廉和腐败行为。深入学习、认真贯彻《公务员法》，大力倡导创新务实、廉洁从政、勤俭节约和艰苦奋斗之风。

各位代表，我们正站在新的历史起点上，肩上的任务光荣而艰巨，让我们紧密团结在以胡锦涛同志为总书记的党中央周围，高举邓小平理论和“三个代表”重要思想旗帜，全面落实科学发展观，在市委的正确领导下，开拓创新，扎实工作，全面完成今年的各项工作任务，为加快建设富裕和谐新贺州而努力奋斗！
